# Reprogramming of Energy Metabolism in Human *PKD1* Polycystic Kidney Disease: A Systems Biology Analysis

**DOI:** 10.3390/ijms25137173

**Published:** 2024-06-29

**Authors:** Xuewen Song, Lauren Pickel, Hoon-Ki Sung, James Scholey, York Pei

**Affiliations:** 1 Division of Nephrology, University Health Network, Toronto, ON M5G 2N2, Canada; 2 Department of Medicine, Division of Nephrology, University of Toronto, Toronto, ON M5S 1A8, Canada; xuewen.song@utoronto.ca (X.S.); james.scholey@uhn.ca (J.S.); 3 Translational Medicine Program, The Hospital for Sick Children, Toronto, ON M5G 1E8, Canada; lauren.pickel@mail.utoronto.ca (L.P.); hoon-ki.sung@sickkids.ca (H.-K.S.); 4 Department of Laboratory Medicine and Pathobiology, University of Toronto, Toronto, ON M5S 1A8, Canada

**Keywords:** ADPKD, renal cysts, global gene profiling, metabolic reprogramming

## Abstract

Multiple alterations of cellular metabolism have been documented in experimental studies of autosomal dominant polycystic kidney disease (ADPKD) and are thought to contribute to its pathogenesis. To elucidate the molecular pathways and transcriptional regulators associated with the metabolic changes of renal cysts in ADPKD, we compared global gene expression data from human *PKD1* renal cysts, minimally cystic tissues (MCT) from the same patients, and healthy human kidney cortical tissue samples. We found gene expression profiles of *PKD1* renal cysts were consistent with the Warburg effect with gene pathway changes favoring increased cellular glucose uptake and lactate production, instead of pyruvate oxidation. Additionally, mitochondrial energy metabolism was globally depressed, associated with downregulation of gene pathways related to fatty acid oxidation (FAO), branched-chain amino acid (BCAA) degradation, the Krebs cycle, and oxidative phosphorylation (OXPHOS) in renal cysts. Activation of mTORC1 and its two target proto-oncogenes, HIF-1α and MYC, was predicted to drive the expression of multiple genes involved in the observed metabolic reprogramming (e.g., *GLUT3*, *HK1/HK2*, *ALDOA*, *ENO2*, *PKM*, *LDHA/LDHB*, *MCT4*, *PDHA1*, *PDK1/3*, *MPC1/2*, *CPT2*, *BCAT1*, *NAMPT*); indeed, their predicted expression patterns were confirmed by our data. Conversely, we found AMPK inhibition was predicted in renal cysts. AMPK inhibition was associated with decreased expression of PGC-1α, a transcriptional coactivator for transcription factors PPARα, ERRα, and ERRγ, all of which play a critical role in regulating oxidative metabolism and mitochondrial biogenesis. These data provide a comprehensive map of metabolic pathway reprogramming in ADPKD and highlight nodes of regulation that may serve as targets for therapeutic intervention.

## 1. Introduction

ADPKD is the most common hereditary kidney disease worldwide with an estimated cumulative lifetime prevalence of ~1 in 1000 [1]. Progressive increase in cyst number and size results in the distortion of normal kidney architecture and ultimately end-stage renal disease in the majority of patients [2]. Mutations of two genes, *PKD1* and *PKD2*, account for 75–85% and 15–25% of the genetically resolved cases, respectively [3,4,5,6]. Recent advances have led to the discovery of multiple therapeutic targets in preclinical studies of ADPKD. Among them, aberrant mTORC1 activation and increased cAMP signaling in cystic tissues are two highly promising pathogenic mechanisms driving cyst growth in ADPKD [7,8]. Both have been experimentally validated and clinically tested as therapeutic targets [7,9,10,11]. However, only vasopressin V2 receptor inhibition by Tolvaptan, which lowers cystic cellular cAMP, has been found to be effective and safe by clinical trials, and has become the first disease-modifying therapy in ADPKD.

As recently reviewed [12,13,14,15,16,17], multiple experimental studies have highlighted a pathogenic role of metabolic reprogramming in ADPKD. Increased aerobic glycolysis [18] and sirtuin 1 (SIRT1) activity [19], reduced AMPK activity [18,20,21,22], mitochondrial dysfunction [18,23,24,25,26,27,28], enhanced reactive oxygen species (ROS) production [27], oxidative stress [24,27,29,30,31,32,33], lipid peroxidation [30,33], defective FAO [34,35], increased glutamine usage [36,37], and arginine auxotrophy [38] have been observed both in vitro and in vivo in animal models of ADPKD or in the tissues of patients with ADPKD. Importantly, targeting metabolic reprogramming defects in ADPKD has been shown to ameliorate cystic disease progression in rodent and non-rodent models [12,13,14,15,16,17].

Repurposing drugs targeting cellular metabolism for the treatment of ADPKD would bypass much of the cost and time associated with novel drug discovery and development [39,40]. For instance, the reliance of *Pkd1* null cells/cystic tissues on glucose for growth and proliferation has led to the use of 2-deoxyglucose as a novel experimental treatment in ADPKD [41,42,43]. Similarly, AMPK is a master metabolic regulator that has been targeted for the treatment of various pathological entities, such as obesity, diabetes, inflammation, and cancer [44,45,46,47]. Accumulating evidence suggests that AMPK activation (using metformin, salsalate, 2-deoxyglucose, or diet) may restore mitochondrial function and slow cystogenesis by inhibiting mTORC1 and the cystic fibrosis transmembrane conductance regulator (CFTR) in the cystic kidney [16,20,22,41,42,43,48,49,50,51,52]. In animal models, the PPARα agonist fenofibrate enhances FAO and attenuates polycystic kidney and liver disease in mice [35], and inhibitors of glutamine metabolism retard disease progression [37,53]. These preclinical findings demonstrate the pivotal importance of better understanding of the interacting metabolic irregularities in ADPKD to identify potential therapeutic targets.

Previously, we performed a systems biology analysis to discover upregulated gene pathways and key transcription factors associated with renal cyst growth in human ADPKD [54]. Of the 637 pathways tested, 212 (128 up- and 84 downregulated) pathways were enriched in renal cysts compared to MCT control. We found that *PKD1* renal cysts displayed a rich network of upregulated signaling pathways for mitogenic responses, including receptor tyrosine kinases (e.g., IGF/IGF1R, FGF/FGFR, EGF/EGFR, VEGF/VEGFR), G-protein-coupled receptors, and intracellular cascades involved in calcium, cAMP and mTORC1 signaling [54]. Here we performed the complementary analysis of gene sets that were downregulated in *PKD1* renal cysts, the majority (77/84) of which were found to be involved in metabolic reprogramming. These data support efforts toward novel therapeutics targeting the key regulators of metabolic reprogramming in ADPKD.

## 2. Results

### 2.1. Metabolic Pathway Analysis of PKD1 Renal Cysts

We used Gene Set Enrichment Analysis (GSEA) to identify dysregulated signaling pathways [55]. The gene sets in the GSEA Molecular Signatures Database (MSigDB) are highly overlapping; Kyoto Encyclopedia of Genes and Genomes (KEGG) is a collection of manually drawn pathways representing experimental knowledge on metabolism and various other functions of the cell; the best organized part of the KEGG pathway database is that of metabolism [56]. In order to reduce the redundancy among the enriched gene sets, we performed GSEA on 186 gene sets from the GSEA C2 KEGG pathway database [55]. At a nominal *p*-value (NOM *p*-value) ≤ 0.01 with a false discovery rate (FDR) ≤ 0.1, we found that 75 pathways were dysregulated (30 up- and 45 downregulated) in the renal cysts (Table 1). Replicating our previous results [54], the upregulated gene sets in *PKD1* renal cysts displayed a rich signature of mitogen-mediated proliferation. By contrast, of the 45 downregulated pathways, 39 represented metabolic pathways or their regulators.

#### 2.1.1. Gene Expression Profiles of Human PKD1 Renal Cysts Are Consistent with the Warburg Effect

**Figure 1:**
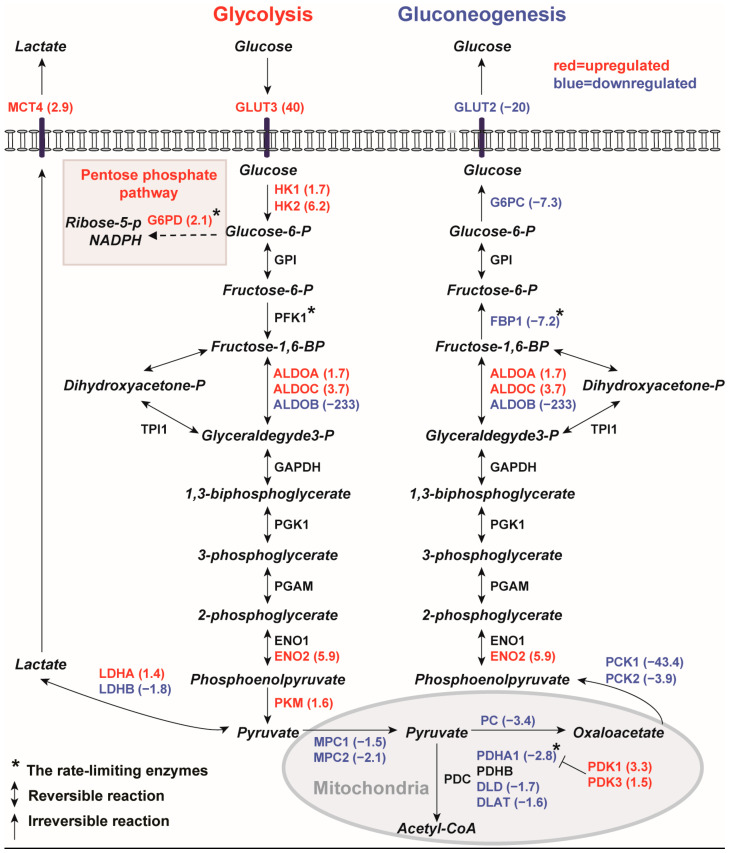
Gene expression profiles of human *PKD1* renal cysts are consistent with the Warburg effect and increased pentose phosphate pathway (PPP) flux. Schematic summary of the upregulation of glycolysis and PPP (left) and downregulation of gluconeogenesis (right) in *PKD1* renal cysts. Upregulated genes are shown in red, and downregulated genes in blue, with mean expression fold changes in brackets. Genes that were not differentially expressed are shown in black. Arrows indicate irreversible enzymatic steps, and bi-directional arrows indicate interconverting reversible reactions determined by substrate concentration. Asterisk * denotes rate-limiting enzymes.

Aerobic glycolysis or the ‘Warburg effect’, a hallmark of cancer or proliferative tissues [18,57], has been observed in animal models and human cystic kidney tissues. KEGG pathway analysis identified glycolysis/gluconeogenesis, pentose phosphate pathway (PPP), and pyruvate metabolism as downregulated in human *PKD1* renal cysts (Table 1). More detailed analysis revealed that key enzymes of gluconeogenesis were highly downregulated, while glycolytic enzymes were moderately upregulated in renal cysts (Figure 1).

To determine whether the gene expression profiles of renal cysts are consistent with the Warburg effect, we first checked changes in the expression of multiple key sequential regulatory points in these pathways. Glucose transporter *GLUT3*, which plays a major role in the enhanced glucose uptake by many cancer cells [58], was upregulated 40× in renal cysts compared to MCT. In addition, enzymes for irreversible steps of glycolysis were upregulated in renal cysts, including hexokinase 1 and 2 (*HK1*, 1.7×; *HK2*, 6.2×), and pyruvate kinase (*PKM*, 1.6×). Genes encoding enzymes for lactate fermentation and export were also upregulated in renal cysts. Lactate dehydrogenase is a tetrameric enzyme consisting of differing ratios of LDHA and LDHB subunits, with LDHA having a higher affinity for pyruvate, and LDHB having a higher affinity for lactate [58]. The upregulation of *LDHA* (1.4×) and downregulation of *LDHB* (−1.8×) in renal cysts suggest increased pyruvate to lactate flux. The carrier that exports glycolysis-derived lactate, *MCT4*, which is predominantly expressed in glycolytic tissues [58], was upregulated (2.9×) in renal cysts (Figure 1, left). On the other hand, the *MPC1* (−1.5×)/*MPC2* (−2.1×) heterodimer responsible for transporting pyruvate into the mitochondria for ATP production [59] was downregulated in renal cysts. Concurrently, multiple genes in the mitochondrial pyruvate dehydrogenase complex (PDC) were downregulated in renal cysts. The PDC acts as a rate-limiting enzyme that catalyzes the irreversible conversion of pyruvate into acetyl coenzyme A (acetyl-CoA), providing the primary link between glycolysis and the Krebs cycle [60]. Downregulated genes include *PDHA1* (−2.8×), *DLAT* (−1.6×), and *DLD* (−1.7×). The activity of the PDC is regulated by the PDHA1 subunit; its phosphorylation by PDH kinases (PDKs) leads to a strong decrease in PDC activity [60]. The upregulation of *PDK1* (3.3×) and *PDK3* (1.5×) in renal cysts suggests the inhibition of PDC activity, and therefore decreased conversion of pyruvate to acetyl-CoA for oxidative metabolism (Figure 1, bottom).

The PPP branches from glycolysis at the first committed step of glucose metabolism to provide the precursors for nucleotide and amino acid biosynthesis. It is the major source of nicotinamide adenine dinucleotide phosphate (NADPH) for the reduction of glutathione (GSH) and fatty acid biosynthesis [58]. Although the PPP pathway was identified as downregulated, we found that *G6PD*, encoding the rate-limiting enzyme for the irreversible oxidative phase of the PPP, was upregulated (2.1×) in renal cysts. The genes contributing to downregulation of the PPP either encoded enzymes of the reversible non-oxidative phase of the PPP, or shared enzymes involved in glycolysis/gluconeogenesis. Among these reversible enzymes shared by the glycolysis/gluconeogenesis and PPP pathways, we found an isoform switch of aldolases in renal cysts. ALDOA has a high affinity for fructose-1,6-BP and favors glycolysis, whereas ALDOB has a higher affinity for glyceraldehyde3-P and dihydroxyacetone phosphate and favors gluconeogenesis [58]. The observed substantial downregulation of *ALDOB* (−233×) and upregulation of *ALDOA* (1.7×) further support increased glycolytic flux in renal cysts. Taken together, these results suggest that, instead of fully oxidizing glucose, the *PKD1* renal cysts shuttle glucose through aerobic glycolysis and the PPP in order to sustain cell growth and proliferation.

#### 2.1.2. Inhibition of Gluconeogenesis Gene Pathway

Gluconeogenesis is the process of generating glucose from non-carbohydrate carbon substrates, such as lactate, glycerol and amino acids [61]. The kidney is the only organ other than the liver able to perform gluconeogenesis [62]. Gluconeogenesis and glycolysis share many reversible enzymes. However, gluconeogenesis uses four distinct reactions to bypass the three metabolically irreversible reactions of glycolysis. The enzymes catalyzing these irreversible reactions are the potential sites for regulatory control [61]. We found that five genes encoding the four enzymes that catalyze the irreversible reactions of gluconeogenesis were all downregulated in renal cysts, including pyruvate carboxylase (*PC*, −3.4×), phosphoenolpyruvate carboxy-kinase (*PCK1*, −43.4×; *PCK2*, −3.9×), fructose 1,2-bisphosphatase (*FBP1*, −7.2×), and glucose 6-phosphate phosphatase (*G6PC*, −7.3×). Notably, FBP1 is the rate-limiting enzyme during gluconeogenesis. In addition, *GLUT2*, a glucose transporter normally enriched in the kidney that is responsible for glucose export, was greatly downregulated (−20×) in renal cysts (Figure 1, right). Overall, these data suggest that gluconeogenesis is downregulated in renal cysts.

#### 2.1.3. Downregulation of Mitochondrial Catalytic Gene Pathways in Renal Cysts

**Figure 2:**
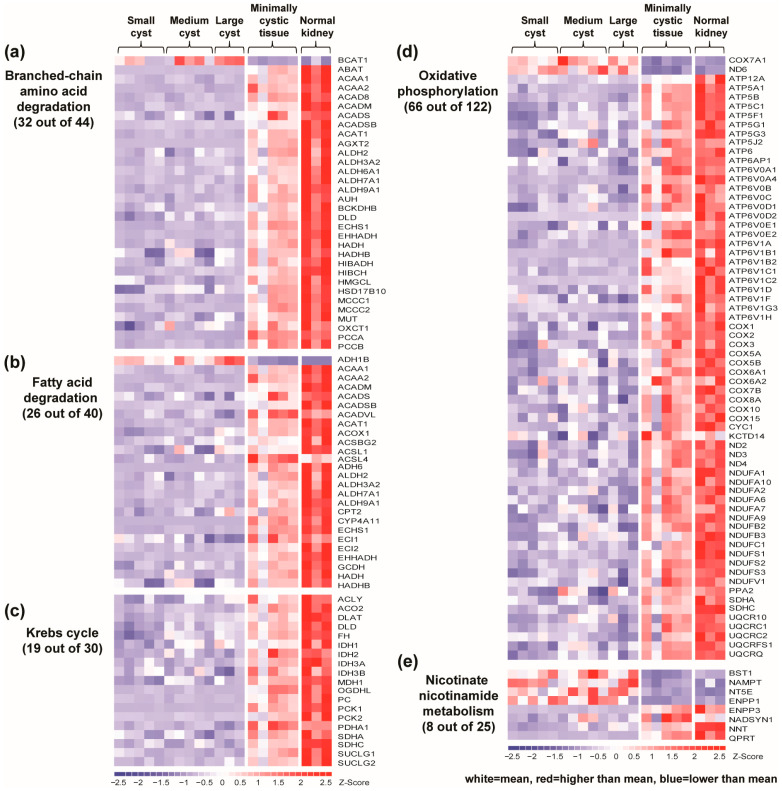
Metabolic reprogramming in human *PKD1* renal cysts. Downregulation of the majority of genes in branched-chain amino acid degradation (**a**), fatty acid degradation (**b**), the Krebs cycle (**c**), and oxidative phosphorylation (**d**) suggests defective mitochondrial oxidative metabolism in *PKD1* renal cysts. (**e**) Upregulation of *NAMPT* and downregulation of *QPRT* suggest renal cysts may favor the salvage over the de novo pathway to produce NAD+. All genes listed in the panels were differentially expressed between the cysts and MCT samples with an FDR ≤ 1%. In the heatmap, each column represents an individual sample, and each row represents the Z-score scaled gene expression levels across all samples; white is the mean Z-score (set to 0), red indicates greater than the mean, and blue less than the mean. Z-scores are computed for individual genes by subtracting the mean and then dividing by the standard deviation.

In normal cells, mitochondrial acetyl-CoA derived from glycolysis, fatty acids, or BCAAs is fed into the Krebs cycle, followed by OXPHOS for high-efficiency ATP generation [63]. Consistent with defective mitochondrial metabolism in ADPKD, 7 of the 10 most downregulated pathways in cystic tissue occur predominantly in the mitochondria. These included BCAA degradation, pyruvate metabolism, fatty acid metabolism, propanoate metabolism, butanoate metabolism, the Krebs cycle, and OXPHOS (Table 1). Most individual genes within these mitochondrial metabolic pathways were also downregulated in renal cysts (Figure 2a–d).

BCAAs (i.e., valine, leucine, and isoleucine) are essential amino acids that play a crucial role in activating mTORC1 [64]. BCAA supplementation has been shown to accelerate the ADPKD progression in mice through mTORC1 and MAPK/ERK activation [65]. Among the downregulated pathways, BCAA degradation was identified as the most downregulated pathway, with 32 differentially expressed genes (31 down, 1 up) in renal cysts (Figure 2a). The one upregulated gene was *BCAT1* (5.3×), which catalyzes the only step in BCAA degradation that occurs outside of the mitochondria, and is the major isoform implicated in cancer growth [64]. In contrast, all 31 downregulated genes encode multiple sequential mitochondrial enzymes in the catabolism of BCAA, suggesting defective mitochondrial BCAA degradation in renal cysts.

Fatty acid metabolism was another highly downregulated pathway, with 26 differentially expressed genes (25 down, 1 up). FAO, which occurs in the mitochondria and peroxisomes, is the preferred energy source for renal tubular epithelial cells [66]. Of interest, all 25 downregulated genes in this pathway encode enzymes in fatty acid degradation. Notably, *CPT2*, encoding one of the rate-limiting enzymes for transferring fatty acids into the mitochondria during FAO, was downregulated (−1.6×) in renal cysts. Concurrently, peroxisome metabolism was also identified among the top downregulated pathways in renal cysts (Table 1). On the other hand, *CD36*, encoding a multifunctional receptor that mediates the binding and cellular uptake of long-chain fatty acids, was greatly upregulated (12.3×), consistent with the upregulation of CD36 in the setting of chronic kidney disease (CKD) [67]. These data suggest increased uptake and reduced catabolism of fatty acids. Together these would cause aberrant intracellular lipid accumulation, which has a demonstrated role in the pathogenesis of kidney injury and fibrosis [34,67,68].

#### 2.1.4. Alteration of GSH Synthesis and GSH-Dependent Antioxidant Response Genes in Renal Cysts

Oxidative damage, as measured by lipid peroxidation [69], has been shown to be greatly elevated in the cystic kidney [30,33], and to drive renal cyst growth by activating the anoctamin 1 (ANO1) [33,70]. Indeed, increased expression of *ANO1* (3.7×) was observed in our *PKD1* renal cysts. Along with evidence of oxidative damage, we found impairment of the GSH-dependent system, which is critical in antioxidant response [71,72]. Our pathway analysis revealed that GSH metabolism, as well as drug metabolism via multiple enzymes, including cytochrome P450, were all downregulated in human *PKD1* renal cysts (Table 1).

**Figure 3:**
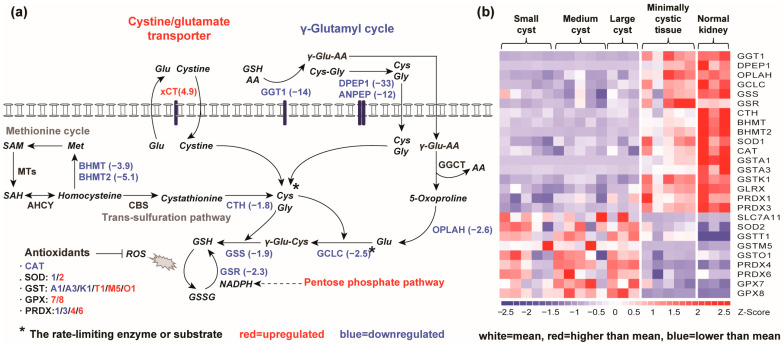
Rewiring of GSH metabolism in human *PKD1* renal cysts. (**a**) Schematic summary of the downregulation of the γ-glutamyl cycle and upregulation of Na+-independent cystine/glutamate antiporter xCT (encoded by *SLC7A11*), which may serve as important sources for maintaining the cysteine pool in *PKD1* renal cysts. NADPH may be resupplied by the reduction of NADP+ via the pentose phosphate pathway. Upregulated genes are shown in red, and downregulated genes in blue, with mean expression fold-changes in brackets. Genes that were not differentially expressed are shown in black. Asterisk * denotes the rate-limiting enzyme or substrate. (**b**) Gene expression profiling showing the differentially expressed genes involved in GSH metabolism in *PKD1* renal cysts. In the heatmap, each column represents an individual sample, and each row represents the Z-score scaled gene expression levels across all samples; white is the mean Z-score (set to 0), red indicates greater than the mean and blue, less than the mean. Z-scores are computed for individual genes by subtracting the mean and then dividing by the standard deviation. Abbreviations: GSH (glutathione); AA (amino acid); Glu (glutamate); Cys (cysteine); Gly (glycine); Met (methionine); ROS (reactive oxygen species); MTs (methyltransferases); SAM (S-adenosylmethionine); SAH (S-adenosylhomocysteine); GSSG (glutathione disulfide); NAPDH (nicotinamide adenine dinucleotide phosphate, reduced); SOD (superoxide dismutase); CAT (catalase); GST (glutathione S-transferase); GPX (glutathione peroxidase); PRDX (peroxiredoxin).

The kidney salvages circulating GSH through the γ-glutamyl cycle, which breaks down extracellular GSH to provide cysteine, the rate-limiting substrate, for intracellular de novo synthesis of GSH [71,73]. We found that multiple genes encoding enzymes in the γ-glutamyl cycle were highly downregulated in renal cysts, including γ-glutamyl transferase (*GGT1*, −14×), dipeptidase 1 (*DPEP1*, −32.5×), aminopeptidase N (*ANPEP*, −12×) and 5-oxoprolinase (*OPLAH*, −2.6×). Levels of GSH biosynthetic enzymes were also downregulated in renal cysts, including the catalytic subunit of the rate-limiting enzyme glutamyl cysteine ligase (*GCLC*, −2.5×), glutathione synthetase (*GSS*, −1.9×), and glutathione reductase (*GSR*, −2.3×) (Figure 3). Aside from the γ-glutamyl cycle, cysteine can be also produced from extracellular cystine through the xCT antiporter encoded by *SLC7A11*, which is known to maintain the cysteine pool in many cancer cells [74,75]. We found that *SLC7A11* was expressed in very low levels in MCT but was upregulated (4.9×) in renal cysts. Cells can also synthesize cysteine de novo from methionine-derived homocysteine using the trans-sulfuration pathway [75]. Expression levels of some enzymes in the trans-sulfuration pathway were reduced, while others were unaltered (Figure 3).

GSH exerts its antioxidant function directly, by interacting with ROS and electrophiles, or by serving as a cofactor for various antioxidant enzymes [69,72]. In renal cysts, we also identified the dysregulation of genes encoding multiple GSH-linked antioxidant enzymes, including superoxide dismutase (*SOD1*, −1.3×; *SOD2*, 1.8×), catalase (*CAT*, −1.6×), and glutathione S-transferase (*GSTA1*, −58×; *GSTA3*, −3.5×; *GSTK1*, −2,2×; *GSTM5*, 2.8×; *GSTO1*, 1.9×), glutathione peroxidase (GPX7 (1.9×), GPX8 (4.1×)), glutaredoxin (*GLRX* (−2.1×)), and peroxiredoxin (*PRDX1*, −1.2×; *PRDX3*, −1.7×; *PRDX4*, 2.8×; *PRDX6*, 1.4×). Of interest, *GSTT1*, encoding glutathione S-transferase theta 1, was consistently overexpressed in both MCT (27×) and renal cysts (33×) relative to normal kidneys. Taken together, these results suggest aberrant GSH synthesis and GSH-dependent antioxidant response in *PKD1* renal cysts.

### 2.2. In Silico Prediction of Key Transcriptional Regulators Based on Differentially Expressed Genes

To discover potential transcriptional regulators responsible for metabolic dysregulation in PKD, we applied our differentially expressed genes with at least 1.5× changes (up: 3142; down: 1690) to Upstream Regulator Analysis (URA) in the Ingenuity^®^ Pathway Analysis (IPA^®^) software (2014 version). URA predicted 102 activated and 48 inhibited transcriptional regulators with z-scores ≥ 2 or ≤−2. Overall, there is excellent concordance between our results from the pathway and URA analyses. The top 50 most activated and 48 most inhibited transcriptional regulators in the renal cysts are shown in Table 2. Many of the predicted transcriptional regulators were differentially expressed in renal cysts compared with MCT.

The most upregulated transcriptional regulators were associated with the activation of TGFβ, growth factor/receptor tyrosine kinase, Wnt/β-catenin, hypoxic, and immune/inflammatory response pathways in *PKD1* renal cysts, consistent with our previous study [54]. In contrast, many of the top inhibited transcriptional regulators were associated with metabolism and development. As expected, URA predicted PKD1 (z-score = −7.8) as the most inhibited protein. Consistent with our previous results [54], hepatocyte nuclear factor family members HNF1α (z-score = −7.3) and HNF4α (z-score = −5), which regulate glucose homeostasis and tissue-specific gene expression, were again predicted to be highly inhibited and both were indeed downregulated in renal cysts. The inhibition of HNF4α also supports experimental work in a *Pkd1* mouse model that identified *Hnf4a* as a key disease modifier [76].

Multifunctional metabolic sensors, including mTORC1, SIRT1, and AMPK, act under a network of cooperative signaling cascades. AMPK is one of the master coordinators of cell energy homeostasis, growth, and metabolism [44,45,46,47]. Of interest, URA predicted the moderate inhibition of AMPKα2 (z-score = −2.1). At the mRNA level, although no significant change was observed in the expression of *PRKKA2* (encoding AMPKα2), we found slightly increased expression of *PRKAA1* (1.5×, encoding AMPKα1) in human *PKD1* renal cysts, consistent with the isoform shift in the catabolic subunit of AMPK from AMPKα2 to AMPKα1 in renal fibrosis [77,78,79]. Other transcriptional regulators that were predicted to be most inhibited in renal cysts included PGC-1α (z-score = −4.9), PPARα (z-score = −3.4) and ERRα (z-score = −3.2). These act under a network of cooperation: AMPK can inhibit mTORC1 and activate PGC-1α, whereas PGC-1α acts as a transcriptional coactivator for PPARα and estrogen-related receptors (e.g., ERRα and ERRγ), which promote the expression of genes in OXPHOS, FAO, the Krebs cycle, and mitochondrial biosynthesis [80,81,82]. Concordantly, the genes encoding PPARα and estrogen-related receptors (*PPARGC1A*, *PPARA*, *ESRRA* and *ESRRG*) were all downregulated in renal cysts.

## 3. Discussion

As one of the most metabolically active organs in the body, the kidney has an abundance of mitochondria to provide sufficient energy for waste filtration, salt-water balance, and electrolyte homeostasis [83,84]. Healthy renal tubular epithelial cells rely on FAO and OXPHOS as their main energy source [66]. In ADPKD, there are reductions in mitochondrial biogenesis, OXPHOS, and FAO, with cells instead relying on aerobic glycolysis (the Warburg effect) to produce energy. Concomitantly, there is decreased AMPK and increased mTORC1 activity.

In this study, we found that gene expression profiles of human *PKD1* renal cysts, regardless of their tubular origins, were consistent with the Warburg effect and had globally depressed mitochondrial oxidative metabolism. Of all pathways involved, mTORC1 and AMPK are two central regulators of energy metabolism, cell growth, and proliferation with opposing effects [44,45,46,47]. mTORC1 integrates signals from growth factors, energy status, oxygen, and amino acid availability to promote anabolic processes and cell growth [44,45,46,47]. mTORC1 also activates two key transcription factors: MYC and HIF-1α [85,86], causing increased expression of genes in aerobic glycolysis (e.g., glucose transporters, glycolytic enzymes) and inhibiting the mitochondrial TCA cycle and OXPHOS. Mitochondrial dysfunction in ADPKD further contributes to reduced FAO and OXPHOS and leads to increased ROS production, causing lipid peroxidation and tissue damage. This is further exacerbated by increased lipid uptake. Activation of ANO1 by lipid peroxidation drives the proliferation and expansion of renal cysts [33,70]. Therefore, restoring mitochondrial homeostasis and function may be beneficial for the treatment of ADPKD.

A target of particular interest is AMPK, a major cellular energy sensor driving catabolic processes, which has received a lot of attention as a treatment target in diseases with underlying metabolic perturbations [44,45,46,47]. AMPK is highly expressed in the kidney and is involved in the regulation of a variety of physiological and pathological processes, including ion transport, podocyte function, renal fibrosis, diabetic renal hypertrophy, and polycystic kidney disease [12,13,16,17,87,88,89]. The AMPK molecule is a heterotrimeric complex composed of a catalytic α subunit, and regulatory β and γ subunits, each of which has multiple isoforms (α1/α2, β1/β2, γ1/γ2/γ3) [44,45,46,47]. In renal fibrosis, AMPKα1 plays a deleterious role, whereas AMPKα2 is protective [77,78,79,90]. Fibrosis and inflammation are common findings in ADPKD, and indeed, we found the gene encoding AMPKα1 to be upregulated in human *PKD1* renal cysts. Given the protective role of AMPKα2 and deleterious role of AMPKα1 in the kidney, we hypothesize that selective activation of AMPKα2-containing isoforms may have the potential to slow ADPKD progression.

An additional function of AMPK is the regulation of PGC-1α by multiple direct and indirect mechanisms [46,47]. As the master regulator of mitochondrial biogenesis, PGC-1α is a transcriptional coactivator interacting with many transcription factors, including PPARα, ERRα, and ERRγ, to stimulate the expression of genes involved in FAO, OXPHOS, and mitochondrial DNA transcription and replication [80,81,82]. Mitochondrial dysfunction along with decreased PGC-1α activity is a common feature of acute kidney injury (AKI) and CKD, and its pharmaceutical activation has reno-protective effects in both [91,92,93]. PGC-1α is also downregulated in murine and human cystic kidney cells and tissues [27,35,50,52,94]. Thus, increasing PGC-1α expression or activity may be a promising approach to restore mitochondrial metabolism and attenuate injury and fibrosis in ADPKD. As an upstream regulator, activation of AMPK would be one method to achieve this.

Regulators of FAO and OXPHOS, both of which are deficient in ADPKD, that were highlighted by our analysis include PPAR*α*, ERRα and ERRγ. PPAR*α* is the master regulator of lipid metabolism, controlling mitochondrial, peroxisomal and microsomal FAO [95]. Notably, fenofibrate, a PPARα agonist, was found to increase FAO and attenuate cystic kidney and liver disease in *Pkd1*^RC/RC^ mice [35]. Both ERRα and ERRγ are orphan nuclear receptors that regulate mitochondrial biogenesis and OXPHOS. Genetic ERRα deficiency leads to abnormal mitochondrial morphology and increases susceptibility to cisplatin-induced AKI in mice [96]. In addition to regulating mitochondrial OXPHOS/FAO functions, ERRγ also cooperates with HNF1β to activate the expression of renal reabsorption genes including *PKD2*; deletion of ERRγ in renal tubular epithelial cells results in renal cysts [97].

In parallel to these metabolic changes, evidence from experimental studies in humans and animals suggests that oxidative stress is increased in ADPKD. The mechanisms underlying oxidative damage remain incompletely understood [24,29,30,31,32,33]. Of interest, GSH depletion with L-buthionine-sulfoximine, a specific inhibitor of γ-glutamyl-cysteine synthetase, caused a marked aggravation of renal cystic disease in a rat model of ADPKD [29]. Our transcriptome profiling in human cysts revealed defective GSH metabolism and a highly downregulated γ-glutamyl cycle. Consistent with our findings, recent integrated transcriptome and metabolome profiling in *Pkd1* mutant mouse kidneys also showed strongly decreased expression of *GGT1* and *DPEP1*, and a striking decrease of multiple γ-glutamyl amino acids, which are the direct products of GGT1 [36]. This indicates that the defective γ-glutamyl cycle pathway in ADPKD is strikingly similar between humans and mice. However, although both *GGT1* and *DPEP1* were found to be greatly inhibited, the levels of cysteine (the direct product of DPEP1), which acts both as a building block for protein translation and as the rate-limiting substrate for GSH synthesis, were not altered, and the levels of GSH were strikingly increased (39×) in *Pkd1* mutant mouse kidneys [36]. Since GSH is an important ROS scavenger, the increased GSH levels could be considered the main strategy used by renal cysts to overcome ROS stress and prevent oxidative stress-induced cell death.

Our data suggest that *Pkd1* mutant cells reprogram their cysteine production to enhance intracellular GSH synthesis through xCT to compensate for the defective γ-glutamyl cycle pathway. The cystine-glutamate antiporter xCT is upregulated in a variety of cancers for cystine uptake and GSH production. Recent studies revealed that xCT also plays a critical role in the glucose and glutamine dependency of cancer cells, and inhibition of xCT activity is emerging as a promising anti-proliferative therapeutic strategy [74,75]. We hypothesize that increased expression of xCT could be an important mechanism of cysteine recruitment for the proliferation of *PKD1* renal cysts.

A previous study revealed that NAD+-dependent enzyme SIRT1 was upregulated and involved in the pathophysiology of a mouse model of ADPKD [19]. Consistent with this, we also found increased expression of *SIRT1* (1.4×) in human *PKD1* renal cysts. In humans, NAD+ is synthesized via two major pathways: via de novo NAD+ biosynthesis and via the NAD+ salvage pathway. Although we found no definitive enrichment of this pathway, we did observe upregulation of *NAMPT* (2.9×) and downregulation of *QPRT* (−12.5×) (Figure 2e), the rate-limiting enzymes in the NAD+ salvage and de novo synthesis pathways, respectively [98]. These data suggest that renal cysts may favor the salvage over the de novo pathway to produce NAD+ for a variety of NAD+-dependent enzymes, including SIRT1.

## 4. Materials and Methods

Renal cysts of different sizes were obtained from 4 *PKD1* polycystic kidneys removed for medical reasons. Small cysts (SC) were defined as less than 1 mL, medium cysts (MC) between 10 and 25 mL and large cysts (LC) greater than 50 mL. Minimally cystic tissue (MCT), which contained no macroscopically observable cysts, was obtained from the same kidney as PKD control tissue. Normal control tissue was obtained from non-cancerous renal cortical tissue from three nephrectomized kidneys with isolated renal cell carcinoma. Using Affymetrix HG-U133 Plus 2.0 arrays (Affymetrix, Santa Clara, CA, USA), global gene profiling was performed on 13 cysts (SC: each pooled from four different SC, *n* = 5; MC, *n* = 5, and LC, *n* = 3), five MCT and three normal renal cortical tissue samples. All the study patients were shown to have *PKD1* by DNA linkage or documentation of a pathogenic mutation identified through DNA sequencing by Athena Diagnostics™ (Marlborough, MA, USA). Informed consent was obtained from all patients and the Institutional Review Board of the hospital where the nephrectomy was performed approved the research protocol used for this study. The surgical technique, RNA extraction, microarray procedure, and bioinformatics analysis used in this study have been described in detail previously (GEO ID: GSE7869) [54].

### 4.1. Pathway Analysis

We used Gene Set Enrichment Analysis (GSEA) (http://software.broadinstitute.org/gsea/index.jsp, accessed on 01 March 2018) to identify dysregulated signaling and metabolic pathways that may modulate renal cyst growth [55]. Before running GSEA, Affymetrix probe sets were collapsed to one gene level by Partek Genomics Suite 6.6 (Partek Inc., Chesterfield, MO, USA) and *t*-test statistics scores were used to create a ranked list of genes of the entire data set (in total, 22,486 unique genes with gene symbols). GSEA was performed using 186 gene sets from the GSEA C2 KEGG pathway database (MSigDB database v6.2 updated July 2018), which has a comprehensive collection of metabolic pathways. We defined overrepresented pathways by a NOM *p*-value ≤ 0.01 with an FDR ≤ 10%.

### 4.2. Upstream Regulator Analysis (URA)

The Upstream Regulator Analysis (URA) feature within the Ingenuity^®^ Pathway Analysis (IPA^®^, QIAGEN, accessed on 16 June 2014) was utilized to infer potential upstream transcriptional regulators influencing gene expression in our microarray dataset. This analysis uses known relationships documented in the Ingenuity^®^ Knowledge Base based on prior scientific findings of the interactions between transcriptional regulators and their target genes. Specifically, the URA algorithm identifies transcriptional regulators whose known target genes are significantly represented in the dataset and assesses the concordance of the observed gene expression changes (upregulation or downregulation) with the expected effects if these regulators were active in order to predict the transcriptional regulatory networks influencing the observed gene expression patterns. For each potential transcriptional regulator, two statistical measures, an activation z-score and an overlap *p*-value, are computed. The z-score and bias-corrected z-score are computed to infer the activation states of upstream regulators. An overlap *p*-value is computed by Fisher’s exact test based on significant overlap between genes in the dataset and known targets regulated by the transcriptional regulator [99]. We used Significance Analysis of Microarrays analysis to identify differentially expressed genes with an FDR ≤ 1% [100]. The top differentially expressed genes with a minimum fold-change of ±1.5 (Cyst vs. MCT) were applied to URA to predict the transcriptional regulators. A bias-corrected z-score ≥ 2 (activated) or ≤−2 (inhibited) was considered significant.

## 5. Conclusions

**Figure 4:**
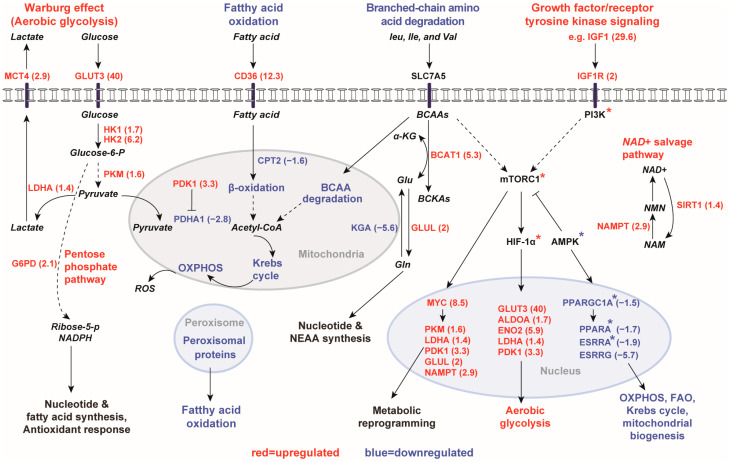
Schematic summary of interrelationships between growth factors and energy sensing pathways in *PKD1* renal cysts. Cysts switch from oxidative metabolism (fatty acid oxidation, branched-chain amino acid degradation, the Krebs cycle, oxidative phosphorylation, and peroxisomal proteins) to aerobic glycolysis to meet their energy needs. The PI3K/Akt pathway is activated upon growth factor/receptor tyrosine kinase stimulation (e.g., IGF1/IGF1R). The mTORC1 pathway integrates signals from growth factor stimulation, amino acid availability, and energy status via AMPK. The oncogenes HIF-1α and MYC together drive the expression of genes promoting aerobic glycolysis and the NAD+ salvage pathway. Upregulated pathways/genes are shown in red, and downregulated pathways/genes in blue, with mean expression fold-changes in brackets. Genes that were not differentially expressed are shown in black. Asterisk * denotes proteins that were predicted to be activated (red) or inhibited (blue) by GSEA or URA. Abbreviations: BCAA (branched-chain amino acid); BCKA (branched-chain α-keto acid); α-KG (α-ketoglutarate); OXPHOS (oxidative phosphorylation); Glu (glutamate); Gln (glutamine); NEAA (non-essential amino acids); ROS (reactive oxygen species); NAD (nicotinamide adenine dinucleotide); NAM (nicotinamide); NMN (nicotinamide mononucleotide).

In conclusion, the present analysis highlights a complex rewiring of energy metabolism in human *PKD1* renal cysts at the level of gene expression. Due to the limited availability of human samples, the lack of protein or metabolite measurements is an important limitation of this work. Future work employing multi-omics will be valuable to confirm the metabolic pathway alterations suggested by the present study. We find that metabolism in cysts is directed toward the generation of metabolic intermediates to support cellular proliferation, rather than efficient extraction of ATP through OXPHOS. We have generated a comprehensive map of key metabolic pathways and regulators altered in *PKD1* renal cysts (Figure 4). Despite the complexity, redundancy, and crosstalk between these pathways, it is conceivable that therapeutic interventions targeting key points of convergence in intracellular signaling cascades may provide broad renal protective effects in ADPKD. For example, our pathway and transcriptional regulator analyses highlighted the importance of AMPK, PGC-1α, PPARα, ERRα, and ERRγ in regulating metabolic reprogramming in ADPKD. These regulators are all highly expressed in the kidney and form an interconnected network. PGC-1α is downstream of the intensively investigated drug target AMPK, while PPARα, ERRα and ERRγ are the downstream targets of PGC-1α. Their expression and/or activity were reduced in renal cysts, in parallel with reduced expression of genes implicated in mitochondrial biogenesis, FAO and OXPHOS. Interventions and drugs that activate an energy-sensing network consisting of these key transcriptional regulators have the potential to inhibit cyst growth.

## Figures and Tables

**Table 1:** Dysregulated KEGG pathways (*n* = 75) in *PKD1* renal cysts (NOM *p* ≤ 0.01 and FDR ≤ 0.1).

Upregulated (*n* = 30)	Size	NES	NOM *p*-val	FDR q-val	Rank by NES
RIBOSOME	81	2.95	0.00	0.000	1
TGF_BETA_SIGNALING_PATHWAY	84	2.44	0.00	0.000	2
SPLICEOSOME	118	2.36	0.00	0.000	3
WNT_SIGNALING_PATHWAY	145	2.19	0.00	0.000	4
NUCLEOTIDE_EXCISION_REPAIR	43	2.00	0.00	0.002	5
FOCAL_ADHESION	197	1.86	0.00	0.013	6
BASAL_CELL_CARCINOMA	53	1.85	0.00	0.012	7
PATHWAYS_IN_CANCER	320	1.85	0.00	0.011	8
ECM_RECEPTOR_INTERACTION	82	1.80	0.00	0.018	9
PATHOGENIC_ESCHERICHIA_COLI_INFECTION	54	1.79	0.00	0.018	10
COLORECTAL_CANCER	62	1.78	0.00	0.018	11
ACUTE_MYELOID_LEUKEMIA	56	1.76	0.00	0.020	12
UBIQUITIN_MEDIATED_PROTEOLYSIS	132	1.76	0.00	0.020	13
MELANOMA	71	1.70	0.01	0.030	14
PROSTATE_CANCER	89	1.70	0.00	0.028	15
RENAL_CELL_CARCINOMA	69	1.68	0.01	0.032	16
MELANOGENESIS	98	1.67	0.00	0.032	17
CELL_CYCLE	123	1.66	0.00	0.035	18
OOCYTE_MEIOSIS	109	1.64	0.00	0.040	19
CHRONIC_MYELOID_LEUKEMIA	72	1.64	0.00	0.040	20
CYTOSOLIC_DNA_SENSING_PATHWAY	54	1.63	0.00	0.040	21
NOD_LIKE_RECEPTOR_SIGNALING_PATHWAY	61	1.63	0.01	0.039	22
REGULATION_OF_ACTIN_CYTOSKELETON	210	1.63	0.00	0.038	23
AXON_GUIDANCE	128	1.62	0.00	0.037	24
JAK_STAT_SIGNALING_PATHWAY	150	1.62	0.00	0.037	25
VIRAL_MYOCARDITIS	67	1.61	0.01	0.039	26
DILATED_CARDIOMYOPATHY	89	1.58	0.01	0.045	27
HYPERTROPHIC_CARDIOMYOPATHY_HCM	82	1.57	0.00	0.048	28
MAPK_SIGNALING_PATHWAY	261	1.57	0.00	0.047	29
CHEMOKINE_SIGNALING_PATHWAY	180	1.47	0.01	0.082	30
**Downregulated (*n* = 45)**	**Size**	**NES**	**NOM** ***p*-val**	**FDR** **q-val**	**Rank** **by NES**
VALINE_LEUCINE_AND_ISOLEUCINE_DEGRADATION *	44	−2.99	0.00	0.000	1
PROPANOATE_METABOLISM *	32	−2.90	0.00	0.000	2
OXIDATIVE_PHOSPHORYLATION *	122	−2.72	0.00	0.000	3
BUTANOATE_METABOLISM *	33	−2.71	0.00	0.000	4
PYRUVATE_METABOLISM *	39	−2.62	0.00	0.000	5
PEROXISOME	77	−2.61	0.00	0.000	6
FATTY_ACID_METABOLISM *	40	−2.60	0.00	0.000	7
PROXIMAL_TUBULE_BICARBONATE_RECLAMATION	23	−2.42	0.00	0.000	8
CITRATE_CYCLE_TCA_CYCLE *	30	−2.41	0.00	0.000	9
ARGININE_AND_PROLINE_METABOLISM	49	−2.39	0.00	0.000	10
BETA_ALANINE_METABOLISM	22	−2.39	0.00	0.000	11
ASCORBATE_AND_ALDARATE_METABOLISM	14	−2.32	0.00	0.000	12
GLYCINE_SERINE_AND_THREONINE_METABOLISM	30	−2.29	0.00	0.000	13
RENIN_ANGIOTENSIN_SYSTEM	17	−2.24	0.00	0.000	14
PPAR_SIGNALING_PATHWAY	67	−2.19	0.00	0.000	15
LYSINE_DEGRADATION	41	−2.17	0.00	0.000	16
GLYCOLYSIS_GLUCONEOGENESIS	60	−2.15	0.00	0.000	17
DRUG_METABOLISM_OTHER_ENZYMES	39	−2.15	0.00	0.000	18
ALANINE_ASPARTATE_AND_GLUTAMATE_METABOLISM	32	−2.12	0.00	0.000	19
FRUCTOSE_AND_MANNOSE_METABOLISM	34	−2.08	0.00	0.001	20
MATURITY_ONSET_DIABETES_OF_THE_YOUNG	24	−1.99	0.00	0.002	21
FOLATE_BIOSYNTHESIS	11	−1.97	0.00	0.002	22
RETINOL_METABOLISM	47	−1.95	0.00	0.002	23
TRYPTOPHAN_METABOLISM	39	−1.95	0.00	0.002	24
TERPENOID_BACKBONE_BIOSYNTHESIS	15	−1.94	0.01	0.002	25
PARKINSONS_DISEASE	118	−1.94	0.00	0.002	26
PENTOSE_AND_GLUCURONATE_INTERCONVERSIONS	16	−1.94	0.00	0.002	27
GLYCEROLIPID_METABOLISM	42	−1.94	0.00	0.002	28
DRUG_METABOLISM_CYTOCHROME_P450	59	−1.94	0.00	0.002	29
LYSOSOME	117	−1.91	0.00	0.003	30
HISTIDINE_METABOLISM	28	−1.90	0.00	0.003	31
HUNTINGTONS_DISEASE	174	−1.88	0.00	0.003	32
LIMONENE_AND_PINENE_DEGRADATION	10	−1.88	0.00	0.003	33
METABOLISM_OF_XENOBIOTICS_BY_CYTOCHROME_P450	57	−1.85	0.00	0.004	34
VIBRIO_CHOLERAE_INFECTION	53	−1.82	0.00	0.006	35
ALZHEIMERS_DISEASE	158	−1.82	0.00	0.006	36
ARACHIDONIC_ACID_METABOLISM	52	−1.81	0.00	0.006	37
STARCH_AND_SUCROSE_METABOLISM	36	−1.81	0.00	0.006	38
PANTOTHENATE_AND_COA_BIOSYNTHESIS	16	−1.78	0.00	0.008	39
PHENYLALANINE_METABOLISM	18	−1.75	0.01	0.010	40
PENTOSE_PHOSPHATE_PATHWAY	26	−1.74	0.00	0.011	41
TYROSINE_METABOLISM	42	−1.74	0.01	0.011	42
STEROID_HORMONE_BIOSYNTHESIS	43	−1.71	0.00	0.013	43
GLUTATHIONE_METABOLISM	47	−1.70	0.00	0.014	44
PORPHYRIN_AND_CHLOROPHYLL_METABOLISM	29	−1.66	0.01	0.019	45

Size is the total number of genes in a given gene set. NES represents degree of enrichment of the gene set at the top or bottom of the ordered gene list. NOM *p*-value measures the significance of NES for a gene set by using permutation testing. The FDR is the estimated probability that a set with a given NES represents a false positive finding. Pathways marked with an asterisk (*) can entirely or mostly be found within mitochondria. Detailed descriptions of each pathway can also be found on the GSEA Molecular Signatures Database (MSigDB) website: http://software.broadinstitute.org/gsea/msigdb/genesets.jsp?collection=CP:KEGG (accessed on 1 March 2018).

**Table 2:** In silico prediction of top activated (*n* = 50) and inhibited upstream regulators (*n* = 48) in *PKD1* renal cysts.

Upstream Regulator	Molecule Type	Predicted Activation State	z-Score	*p*-Value of Overlap
**Activated** **(z-score ≥ 2)**				
TGFB1	growth factor	Activated	5.99	4.73 × 10^−18^
NUPR1	transcription regulator	Activated	5.91	1.32 × 10^−1^
Tgf beta	growth factor	Activated	4.50	2.81 × 10^−5^
IL1B	cytokine	Activated	4.39	2.34 × 10^−7^
IL6	cytokine	Activated	3.82	3.60 × 10^−2^
NR0B2	ligand-dependent nuclear receptor	Activated	3.82	2.01 × 10^−3^
SMAD4	transcription regulator	Activated	3.68	1.13 × 10^−3^
TGFBR2	kinase	Activated	3.67	9.54 × 10^−5^
Vegf	growth factor	Activated	3.59	1.71 × 10^−4^
WNT1	cytokine	Activated	3.59	1.97 × 10^−5^
TGFB3	growth factor	Activated	3.51	1.45 × 10^−9^
F2	peptidase	Activated	3.49	1.03 × 10^−5^
TNF	cytokine	Activated	3.48	1.54 × 10^−11^
TGFA	growth factor	Activated	3.33	2.24 × 10^−1^
LDL	complex	Activated	3.32	1.99 × 10^−1^
IL17A	cytokine	Activated	3.18	8.81 × 10^−2^
SRF	transcription regulator	Activated	3.17	2.43 × 10^−2^
IL1A	cytokine	Activated	3.15	1.02 × 10^−2^
EDN1	cytokine	Activated	3.12	7.12 × 10^−2^
MKL1	transcription regulator	Activated	3.10	5.94 × 10^−2^
STAT4	transcription regulator	Activated	3.04	2.04 × 10^−7^
SMAD3	transcription regulator	Activated	3.01	6.54 × 10^−4^
EGF	growth factor	Activated	2.94	3.73 × 10^−5^
P38 MAPK	mitogen-activated protein kinase	Activated	2.82	4.94 × 10^−3^
CSF3	cytokine	Activated	2.82	5.17 × 10^−1^
FOXL2	transcription regulator	Activated	2.77	4.34 × 10^−1^
MTPN	transcription regulator	Activated	2.75	1.05 × 10^−4^
IFNG	cytokine	Activated	2.71	3.54 × 10^−5^
IGF2BP1	translation regulator	Activated	2.71	3.43 × 10^−5^
HTT	transcription regulator	Activated	2.68	4.33 × 10^−5^
TGFBR1	kinase	Activated	2.67	2.17 × 10^−5^
HGF	growth factor	Activated	2.66	1.37 × 10^−4^
C5	cytokine	Activated	2.66	1.00 × 10^0^
STAT3	transcription regulator	Activated	2.62	5.35 × 10^−2^
OSM	cytokine	Activated	2.59	3.47 × 10^−9^
F7	peptidase	Activated	2.59	1.65 × 10^−5^
CYP1B1	enzyme	Activated	2.56	2.38 × 10^−4^
Cg	complex	Activated	2.56	3.21 × 10^−8^
IRF8	transcription regulator	Activated	2.55	1.00 × 10^0^
MAP2K1/2	MEK/ERK	Activated	2.55	6.69 × 10^−3^
HIF1A	transcription regulator	Activated	2.55	7.28 × 10^−5^
GDF9	growth factor	Activated	2.55	5.25 × 10^−3^
SMAD2	transcription regulator	Activated	2.53	1.00 × 10^0^
NRG1	growth factor	Activated	2.53	1.74 × 10^−2^
CTNNB1	transcription regulator	Activated	2.50	9.74 × 10^−11^
MAP3K1	kinase	Activated	2.49	1.32 × 10^−1^
CSF1	cytokine	Activated	2.48	4.49 × 10^−1^
PDGF BB	complex	Activated	2.47	9.83 × 10^−17^
SRC	kinase	Activated	2.45	8.64 × 10^−4^
ADAM17	peptidase	Activated	2.43	2.63 × 10^−1^
**Inhibited** **(z-score ≤ -2)**				
PKD1	ion channel	Inhibited	−7.82	3.64 × 10^−27^
HNF1A	transcription regulator	Inhibited	−7.29	2.31 × 10^−6^
LHX1	transcription regulator	Inhibited	−7.07	2.01 × 10^−14^
PXR ligand-PXR-Retinoic acid-RXR	complex	Inhibited	−5.34	8.61 × 10^−4^
HNF4A	transcription regulator	Inhibited	−4.97	1.10 × 10^−8^
PPARGC1A	transcription regulator	Inhibited	−4.88	1.51 × 10^−2^
INSR	kinase	Inhibited	−4.54	3.69 × 10^−3^
Alpha catenin	group	Inhibited	−4.16	1.24 × 10^−7^
Ncoa-Nr1i2-Rxra	complex	Inhibited	−4.11	2.81 × 10^−4^
CAR ligand-CAR-Retinoic acid-RXR	complex	Inhibited	−4.07	3.38 × 10^−3^
Ncoa-Nr1i3-Rxra	complex	Inhibited	−3.68	1.01 × 10^−2^
HNF4 dimer	complex	Inhibited	−3.64	1.01 × 10^−2^
AHR	ligand-dependent nuclear receptor	Inhibited	−3.51	4.40 × 10^−12^
WISP2	growth factor	Inhibited	−3.45	5.90 × 10^−3^
PPARA	ligand-dependent nuclear receptor	Inhibited	−3.44	1.33 × 10^−3^
FOXA2	transcription regulator	Inhibited	−3.41	1.86 × 10^−1^
estrogen receptor	group	Inhibited	−3.25	3.21 × 10^−9^
ESRRA	ligand-dependent nuclear receptor	Inhibited	−3.23	1.36 × 10^−1^
SOX2	transcription regulator	Inhibited	−3.22	5.04 × 10^−4^
NKX2-1	transcription regulator	Inhibited	−3.20	8.61 × 10^−3^
SGK1	kinase	Inhibited	−3.16	2.07 × 10^−1^
FOXA3	transcription regulator	Inhibited	−3.15	7.92 × 10^−3^
DICER1	enzyme	Inhibited	−2.97	3.94 × 10^−3^
POU3F3	transcription regulator	Inhibited	−2.91	3.95 × 10^−2^
RXRA	ligand-dependent nuclear receptor	Inhibited	−2.86	7.54 × 10^−4^
FOXI1	transcription regulator	Inhibited	−2.84	1.52 × 10^−3^
SMAD7	transcription regulator	Inhibited	−2.82	3.44 × 10^−7^
FGF21	growth factor	Inhibited	−2.71	1.25 × 10^−1^
Immunoglobulin	complex	Inhibited	−2.67	3.73 × 10^−1^
ALDH1A2	enzyme	Inhibited	−2.67	1.73 × 10^−4^
KRAS	enzyme	Inhibited	−2.63	8.58 × 10^−8^
NR4A3	ligand-dependent nuclear receptor	Inhibited	−2.58	3.87 × 10^−1^
DKK1	growth factor	Inhibited	−2.40	1.29 × 10^−3^
MAX	transcription regulator	Inhibited	−2.35	1.42 × 10^−2^
KLF2	transcription regulator	Inhibited	−2.32	3.91 × 10^−2^
CFTR	ion channel	Inhibited	−2.32	2.12 × 10^−3^
GSK3B	kinase	Inhibited	−2.29	3.75 × 10^−1^
NOG	growth factor	Inhibited	−2.29	2.88 × 10^−1^
PPIF	enzyme	Inhibited	−2.24	5.10 × 10^−1^
SPDEF	transcription regulator	Inhibited	−2.20	2.17 × 10^−5^
DACH1	transcription regulator	Inhibited	−2.15	3.50 × 10^−3^
AMPKα2	kinase	Inhibited	−2.13	2.97 × 10^−1^
PTPN1	phosphatase	Inhibited	−2.10	1.00 × 10^0^
SPTLC2	enzyme	Inhibited	−2.10	3.88 × 10^−2^
Laminin	complex	Inhibited	−2.07	5.25 × 10^−4^
INHA	growth factor	Inhibited	−2.05	1.92 × 10^−8^
KDM1A	enzyme	Inhibited	−2.03	1.00 × 10^0^
ERP29	transporter	Inhibited	−2.00	1.48 × 10^−1^

The bias-corrected z-score is used to infer the activation states of upstream regulators. It is calculated from the proportions of genes that are differentially regulated in an expected direction based on the known interactions between the regulator and the genes present in the Ingenuity database. z-scores ≥ 2 or ≤−2 are considered to be either activated or inhibited, respectively. The *p*-value of overlap is the calculated statistical significance of overlap between genes from the dataset and genes that are known to be regulated by the regulator using Fisher’s exact test. Gene expression direction is not taken into account for this calculation.

## Data Availability

Microarray data are available in Gene Expression Omnibus (GEO, http://www.ncbi.nlm.nih.gov/geo/) (ID: GSE7869).

## Abbreviations

ADPKD (autosomal dominant polycystic kidney disease); FAO (fatty acid oxidation); BCAA (branched-chain amino acid); OXPHOS (oxidative phosphorylation); GSH (glutathione); ROS (reactive oxygen species); GSEA (gene set enrichment analysis); MSigDB (Molecular Signatures Database); KEGG (Kyoto Encyclopedia of Genes and Genomes); NOM *p*-value (nominal *p*-value); FDR (false discovery rate); PPP (pentose phosphate pathway); NAPDH (nicotinamide adenine dinucleotide phosphate, reduced); PDC (pyruvate dehydrogenase complex); PDK (PDH kinase); acetyl-CoA (acetyl coenzyme A); BCKA (branched-chain α-keto acid); α-KG (α-ketoglutarate); NAD (nicotinamide adenine dinucleotide); NAM (nicotinamide); NMN (nicotinamide mononucleotide); AA (amino acid); Glu (glutamate); Gln (glutamine); Cys (cysteine); Gly (glycine); Met (methionine); GSSG (glutathione disulfide); MTs (methyltransferases); SAM (S-adenosylmethionine); SAH (S-adenosylhomocysteine); SOD (superoxide dismutase); CAT (catalase); GST (glutathione S-transferase); GPX (glutathione peroxidase); PRDX (peroxiredoxin); NEAA (non-essential amino acid); IPA^®^ (Ingenuity^®^ Pathway Analysis); URA (Upstream Regulator Analysis); NES (normalized enrichment score); AKI (acute kidney injury); CKD (chronic kidney disease); MCT (minimally cystic tissue).
